# Supplementary material for: Characterization of a novel variant in the HR1 domain of MFN2 in a patient with ataxia, optic atrophy and sensorineural hearing loss
Source: F1000Res. 2022 Sep 2;10:606. Originally published 2021 Jul 19. [Version 2] doi: 10.12688/f1000research.53230.2 (PMC10808857; doi:10.12688/f1000research.53230.2)
Supplement: Peer review history

# Reviewer report: Reviewer response for version 2

**DOI:** 10.5256/f1000research.136924.r218485

**Published:** 2024-01-24

**Authors:** Antonietta Franco

**Affiliations:** 1 University of Washington, Seattle, Washington, USA

Sharma *et al.*, characterized a new novel variant in MFN2 and expanded the phenotypic presentation of *MFN2* variants. Moreover they added more important information related to mitochondria fusion and mitochondria mtDNA maintenance, opening new investigation field in mitochondria DNA mutation. 

 The authors improved the current version with no request to add further.

Is the work clearly and accurately presented and does it cite the current literature?

Yes

If applicable, is the statistical analysis and its interpretation appropriate?

Yes

Are all the source data underlying the results available to ensure full reproducibility?

Yes

Is the study design appropriate and is the work technically sound?

Yes

Are the conclusions drawn adequately supported by the results?

Yes

Are sufficient details of methods and analysis provided to allow replication by others?

Yes

Reviewer Expertise:

Mitochondria quality control

I confirm that I have read this submission and believe that I have an appropriate level of expertise to confirm that it is of an acceptable scientific standard.

# Reviewer report: Reviewer response for version 2

**DOI:** 10.5256/f1000research.136924.r149306

**Published:** 2022-09-06

**Authors:** Daniele Ghezzi, Alessia Nasca

**Affiliations:** 1 Unit of Medical Genetics and Neurogenetics, Fondazione IRCCS Istituto Neurologico Carlo Besta, Milan, Italy; 2 DEPT, University of Milan, Milan, Italy

Version 2 of the manuscript has been greatly improved, with additional experimental findings and a better discussion.

 The only limitation concerns the limited number of cell lines analysed: one single line for control fibroblasts, and the absence of additional lines carrying other *MFN2* mutations (useful to define what defects are specific for each mutation).

Is the work clearly and accurately presented and does it cite the current literature?

Yes

If applicable, is the statistical analysis and its interpretation appropriate?

I cannot comment. A qualified statistician is required.

Are all the source data underlying the results available to ensure full reproducibility?

Yes

Is the study design appropriate and is the work technically sound?

Yes

Are the conclusions drawn adequately supported by the results?

Partly

Are sufficient details of methods and analysis provided to allow replication by others?

Yes

Reviewer Expertise:

Neurogenetics, mitochondrial diseases

We confirm that we have read this submission and believe that we have an appropriate level of expertise to confirm that it is of an acceptable scientific standard.

# Reviewer report: Reviewer response for version 1

**DOI:** 10.5256/f1000research.56591.r98902

**Published:** 2021-11-19

**Authors:** Daniele Ghezzi, Alessia Nasca

**Affiliations:** 1 Unit of Medical Genetics and Neurogenetics, Fondazione IRCCS Istituto Neurologico Carlo Besta, Milan, Italy; 2 DEPT, University of Milan, Milan, Italy

The manuscript by Sharma et al. describes the studies on a novel *MFN2 *variant (p. D414V), identified in a patient with ataxia, optic atrophy and deafness.

 Our first objection concerns the phenotypic expansion to include cerebellar ataxia. It has been already reported in subjects with *MFN2* variants (Rouzier *et al.* 2012 ^1^), and, in fact, this gene was present in the panel for ataxic syndromes used by the authors.

 As a general thought, in a single patient harboring a putative - causative homozygous variant (hence with high probability of consanguinity for the parents) and presenting with atypical features, it is important to consider the possibility of dual molecular diagnosis to explain the unexpected clinical signs. Even deep functional studies cannot demonstrate that the whole set of clinical presentations are due to a single mutation. Only additional cases with the same phenotype can confirm the genotype-phenotype link.

 Indeed, even with WES, some mutations (e.g. intronic ones) can be missed. Moreover, it is not clear if the sequencing of the whole mtDNA has been performed.

 Regarding the statement that “These findings raise the possibility that impaired fusion does not lead to peripheral neuropathy per se”, it is worthy to mention that mutations in genes involved in mitochondrial fission, i.e. the opposite process to fusion, may be associated with peripheral neuropathy (e.g. *MFF*) or optic atrophy (e.g. *DNM1L*). It is possible to speculate that impairment of the fine regulation of mitodynamics, in either direction, contributes to the pathological phenotype.

 The characterization in patient’s fibroblasts is very detailed and with interesting findings, but also with some flaws.

1. It would have been extremely useful to perform the same experimental set in fibroblasts carrying other *MFN2* mutations, in order to define what defects are specific for each mutation.
2. The strong reduction in oxygen consumption is unexpected since most of the cases with mutations in mitodynamics genes have poor or no biochemical defect. The very limited decrease in mtDNA amount cannot explain this result. Were the control cell lines aged-matched? and at the same sub-cultural passage?
3. For mtDNA copy number evaluation, authors use 18S as nuclear-encoded housekeeping gene. The small subunit 18S rRNA gene is one of the most frequently used genes in phylogenetic studies. In general, rRNA gene are organized in cluster, and each cluster consists of multiple rDNA repeat units that vary in number among individuals and chromosomes, so their repetitive arrangement within the genome provides excessive amounts of template DNA for PCR with total diploid copy number estimates ranging from 60 to >800 repeat units in a human genome. Instead, single copy genes (e.g. RNASEH, β2M) are widely used control for qRT-PCR analyses because of its invariant expression across tissues, cells, and subjects.
4. Analysis on the protein is missing. There are numerous well-functioning antibodies against MFN2 and given the availability of the patient's fibroblasts it would be useful to check the amount of the protein.
5. Experiments for mito-network evaluation were conducted in fixed cells. The fixing process could alter the result; it would be recommended to perform these experiments using live imaging. Furthermore, since fragmented mitochondrial networks is not evident (by looking at the images), we suggest to try to stress mitochondrial dynamics in fibroblasts (e.g. by culturing them in galactose) to see if there is any clear alteration in morphology.

Is the work clearly and accurately presented and does it cite the current literature?

Yes

If applicable, is the statistical analysis and its interpretation appropriate?

I cannot comment. A qualified statistician is required.

Are all the source data underlying the results available to ensure full reproducibility?

Yes

Is the study design appropriate and is the work technically sound?

Yes

Are the conclusions drawn adequately supported by the results?

Partly

Are sufficient details of methods and analysis provided to allow replication by others?

Yes

Reviewer Expertise:

Neurogenetics, mitochondrial diseases

We confirm that we have read this submission and believe that we have an appropriate level of expertise to confirm that it is of an acceptable scientific standard, however we have significant reservations, as outlined above.

## References

[1] : The MFN2 gene is responsible for mitochondrial DNA instability and optic atrophy 'plus' phenotype. *Brain* .2012;135(Pt 1) : 10.1093/brain/awr323 23-34 10.1093/brain/awr323 22189565

# Response

**Published:** 2022-07-30

**Authors:** Timothy Shutt

**Affiliations:** University of Calgary, Canada

*>We would like to thank the reviewers for their interest in the manuscript and their suggestions, which we have taken to heart and have addressed to the best of our ability. We would also like to apologize for the extended period of time for the revisions. The first author left the lab shortly after the paper was initially submitted, and it took some time to get a new person up and running on the project after the reviews were all received. In addition to several clarifications in the text, the revised version of the manuscript contains new data showing alterations to the expression of mitochondrial fusion proteins, as well as new mtDNA copy number data normalized to B-tubulin. We hope that the revisions answer the questions and issues raised by the reviewers. Below are the specific point by point response to the reviews.*

 Reviewer Expertise

 I have worked on mitochondrial genetics for 3 decades, and now on mitochondrial dynamics and mitophagy.

 The manuscript by Sharma et al. describes the studies on a novel *MFN2 *variant (p. D414V), identified in a patient with ataxia, optic atrophy and deafness.

 Our first objection concerns the phenotypic expansion to include cerebellar ataxia. It has been already reported in subjects with *MFN2* variants (Rouzier *et al.* 2012 ^1^ ), and, in fact, this gene was present in the panel for ataxic syndromes used by the authors.

*>Thank you for bringing this important article to our attention. This work characterises in detail a family with a heterozygous variant in MFN2, c.629A>T (p.D210V). The family is described to have variable clinical severity, penetrance, and optic atrophy. The index case is reported as having “sensory and cerebellar ataxia” without providing details about the clinical examination. Neuroimaging showed cerebral atrophy but there was no mention of cerebellar structural change and representative images did not include the cerebellum. Eleven other family members were described as having sensory ataxic neuropathy, but not cerebellar ataxia. This being the case, it seems the phenotype being described with the D210V substitution is sensory ataxia, which implies a different anatomic localisation than cerebellar ataxia. It does remain possible cerebellar ataxia was present in the index case, but the examination findings were not described, MRI findings did not show cerebellar abnormalities, and none of the 11 other affected members in that pedigree had cerebellar findings. In contrast, our case is the first to demonstrate clear-cut cerebellar ataxia, with cerebellar examination findings, absence of major sensory findings, and presence of cerebellar atrophy on neuroimaging. To provide this important additional information we bring attention to this article in the introduction. We have also included additional text at the beginning of the discussion:*

 “ *There is one described report of an MFN2 patient having neuropathy with sensory ataxia and optic atrophy. The index case was actually described as having “sensory and cerebellar ataxia” however the presence of cerebellar dysfunction in this case is uncertain because physical findings were not described, the MRI did not have cerebellar abnormalities, and none of the other 11 cases in the pedigree were considered to have cerebellar findings. In contrast, our case is the first to demonstrate clear-cut cerebellar ataxia, with cerebellar examination findings, absence of major sensory findings, and presence of cerebellar atrophy on neuroimaging.”*

*With regard to MFN2 being present on the ataxia panel, genetic testing companies will frequently include a broad list of genes associated with the relevant phenotypes or potential diagnostic mimickers. The genetic testing company does not publish justifications for the content of their panels, but we think it is likely MFN2 was included because of the report by Rouzier and colleagues showing sensory ataxia in several patients, and sensory ataxia can be difficult to distinguish clinically from cerebellar ataxia in some cases.*

 As a general thought, in a single patient harboring a putative - causative homozygous variant (hence with high probability of consanguinity for the parents) and presenting with atypical features, it is important to consider the possibility of dual molecular diagnosis to explain the unexpected clinical signs. Even deep functional studies cannot demonstrate that the whole set of clinical presentations are due to a single mutation. Only additional cases with the same phenotype can confirm the genotype-phenotype link.

*>Thank you for raising this important and very thoughtful point. It is agreed that consanguinity is likely for the parents, although not proven. The possibility of a dual molecular diagnosis was considered as a possibility early on, and this was the main reason that we proceeded with exome sequencing after identifying the MFN2 variant with panel testing. Findings from exome sequencing did not identify any additional diagnosis. We agree that definitively associating this complex phenotype to this homozygous variant will only be possible by identifying additional cases. In the meantime, we believe that the extensive functional characterisation provides strong evidence connecting this variant to the clinical presentation. To address this comment the discussion includes the following comment:*

*“However, as only a single D414V patient has been described to date, we cannot infer definitively on genotype/phenotype correlation.”*

 Indeed, even with WES, some mutations (e.g. intronic ones) can be missed. Moreover, it is not clear if the sequencing of the whole mtDNA has been performed.

*>Exome sequencing has coverage for intron-exon boundaries but it is agreed that deeper intronic variants would not be detectable. The patient’s muscle biopsy was done when he lived in a different country, so we did not have access to muscle tissues for mtDNA sequencing. Nonetheless, we did attempt to identify mtDNA variants from the off-target reads of our exome sequencing. In general, this approach is only considered accurate if read depth is 20X or greater (PMID: 24901348), which was the case for only 6% of the mitochondrial genome. Within the limitations of this approach, we did not identify pathogenic variants. We have updated the manuscript with this information and the caveat in limitations that a mtDNA variant remains possible. Added to discussion:*

*“Other limitations of this study include the possibility that another clinically relevant intronic variant was missed due to the use of exome sequencing. We did not perform mitochondrial genome sequencing (and coverage of mtDNA from off-target reads was very low in our exome analysis), so it is also possible a relevant mtDNA variant was missed.”*

 Regarding the statement that “These findings raise the possibility that impaired fusion does not lead to peripheral neuropathy per se”, it is worthy to mention that mutations in genes involved in mitochondrial fission, i.e. the opposite process to fusion, may be associated with peripheral neuropathy (e.g. *MFF*) or optic atrophy (e.g. *DNM1L*). It is possible to speculate that impairment of the fine regulation of mitodynamics, in either direction, contributes to the pathological phenotype.

*>As suggested, we have added this notion to the revised introduction.*

 The characterization in patient’s fibroblasts is very detailed and with interesting findings, but also with some flaws.

1. It would have been extremely useful to perform the same experimental set in fibroblasts carrying other *MFN2* mutations, in order to define what defects are specific for each mutation.

*>We agree with the reviewer that looking at other MFN2 variants would be of value. Unfortunately, we don’t have access to other patient cell lines carrying ‘classic’ MFN2 variants to make such comparisons. Notably, this is an issue that is also present in many other reports of MFN2 variants, which often only look at one or two molecular functions (if that) for a single MFN2 variant. Notably, since the initial submission of this manuscript, we have published a review paper that tries to summarize what is known about many different MFN2 variants with respect to the various MFN2 functions (PMID: 35399520).*

  

1. The strong reduction in oxygen consumption is unexpected since most of the cases with mutations in mitodynamics genes have poor or no biochemical defect. The very limited decrease in mtDNA amount cannot explain this result. Were the control cell lines aged-matched? and at the same sub-cultural passage?

*>Yes, the control cells were age-matched and analyzed at the same passage. We note that while reduced oxygen consumption is not always observed with MFN2 variants, there are several examples (also linked to mtDNA depletion) where this is the case (reviewed in PMID: 35399520). We also note that our new data suggest a significant decrease in mitochondrial abundance, which likely contributes to the reduced oxygen consumption.*

1. For mtDNA copy number evaluation, authors use 18S as nuclear-encoded housekeeping gene. The small subunit 18S rRNA gene is one of the most frequently used genes in phylogenetic studies. In general, rRNA gene are organized in cluster, and each cluster consists of multiple rDNA repeat units that vary in number among individuals and chromosomes, so their repetitive arrangement within the genome provides excessive amounts of template DNA for PCR with total diploid copy number estimates ranging from 60 to >800 repeat units in a human genome. Instead, single copy genes (e.g. RNASEH, β2M) are widely used control for qRT-PCR analyses because of its invariant expression across tissues, cells, and subjects.

*>We have reanalyzed the mtDNA copy number compared to both the multicopy 18S and the single copy B-tubulin (new fig 3D). Notably, copy number is decreased significant with respect to for both controls.*

1. Analysis on the protein is missing. There are numerous well-functioning antibodies against MFN2 and given the availability of the patient's fibroblasts it would be useful to check the amount of the protein.

*>We investigated the levels of MFN2 in our patient fibroblasts, and discovered some interesting differences. Notably, we see decreased protein levels for MFN2, as well as several other mitochondrial proteins, suggesting that overall mitochondrial abundance is reduced in the patient cells. These new data and a discussion of these novel results has been added to the revised manuscript.*

1. Experiments for mito-network evaluation were conducted in fixed cells. The fixing process could alter the result; it would be recommended to perform these experiments using live imaging. Furthermore, since fragmented mitochondrial networks is not evident (by looking at the images), we suggest to try to stress mitochondrial dynamics in fibroblasts (e.g. by culturing them in galactose) to see if there is any clear alteration in morphology.

*>We have extensive experience in imaging mitochondria, and are confident that our fixation methods, which are widely used in the field for this purpose, do not introduce artefacts. There are also potential artefactual issues with live-cell imaging, as lasers can induce mitochondrial stress, so there is no perfect way to image mitochondrial networks. Our quantitative analysis of the mitochondrial size does in fact show significant differences between control on patient mitochondrial networks when many cells are examined. Though they may not seem fragmented to the reviewer, these differences are evident to the practiced eye in the representative images. As we already see significant differences in the cells when grown in normal glucose containing media, we did not culture our cells in galactose.*

# Reviewer report: Reviewer response for version 1

**DOI:** 10.5256/f1000research.56591.r89928

**Published:** 2021-08-12

**Authors:** Joanna Poulton, Nynke van Polanen

**Affiliations:** 1 Nuffield Department of Women's and Reproductive Health, University of Oxford, Oxford, UK; 2 University of Oxford, Oxford, UK

Thank you for asking me to review this paper characterizing a mutation in MFN2 that results in an unusual phenotype. I think that this paper is ready for indexing provided they can confirm some additional details including the number of controls studied.

**Main points:**

 1. Neither the methods nor the results clearly spell out the N for each experiment. Figure 2B suggests that data was generated from 24 control and 27 patient fibroblasts.  How many controls were used?  The study would not be ready for publication if they only used one control.  They should state how many repetitions of each of the experiments there were (their single statement, “…three independent biological replicates” on page 6 is not sufficiently detailed).  These additional data would provide important support for the findings. Even though the findings are relevant and interesting, that only one patient has been used should be stated more clearly along with the need to study more patients/relative with the novel D414V MFN2 variant.

 2. The loss of mitochondria ER contacts (MERCs) is very beautiful and interesting.  This may well be linked to the slight reduction in mitochondrial DNA copy number and in respiratory chain function. The authors discuss the hypothesis that mtDNA replication is impaired in MFN2 because of the effect of excessive mitochondrial fragmentation on the distribution of the replication machinery (ref 19).  However, they barely discuss two other factors that are relevant to mtDNA replication: mitochondrial dynamics affect both nucleoid division and mitophagy (and hence mtDNA turnover).  Interactions with the ER are thought to license mtDNA replication as well as initiating nucleoid division (ref 30) so having a reduced MERC area, and potentially having a smaller midzone (Kleele *et al. *(2021 ^1^)) could have an important effect. Impaired mitochondrial fusion may increase (Liao *et al*. (2017 ^2^)) or decrease (Sebastián *et al*.(2016 ^3^)) mitophagy in impaired MFN2 expression. Increased mitophagy could decrease mtDNA copy number and slow recovery from mtDNA depletion (ref 19).

 3. The decrease in lipid droplets is interesting and I think novel in MFN2 mutants. Examining the distribution of lipid droplets in the form of distance to the nucleus is also a nice addition to the data. They should mention that (i) the exact molecular mechanisms how MFN2 maintains lipid homeostasis is unknown (ii) perilipin 1 much more abundant in adipose tissue (ref 34) than fibroblasts. From the discussion it is not clear how perilipins can explain the altered distribution found in the fibroblasts in the present paper. The lipid droplets in figure 5 could follow either (i) the mitochondrial network which seems also more perinuclear in the representative figure or (ii) the microtubular network which could be clarified by drug treatments such as nocodazole.

 4. Was the number of mitochondrial nucleoids or lipid droplets corrected for the cell size? Did the authors also measure the size of the lipid droplets in these cells?

 5. The patient’s presentation is interesting: impaired vision, poor motor function and deafness. Of these, unusual features included early loss of vision, cerebellar ataxia, and brain MRI abnormalities.  However, some basic clinical details are missing.

- a) No segregation analysis is presented, presumably his parents may be no longer available. It would be useful to support recessive inheritance by identifying an asymptomatic individual in the pedigree who is heterozygous for the same mutation.
- b)  Analysis of skeletal muscle for mitochondrial DNA copy number and variable deletions.  Muscle is more relevant than these data that they present in fibroblasts, and should be available because the patient had a muscle biopsy.

6. The authors present a nice visualization of mitochondrial fusion. As MFN1 is also important in mitochondrial fusion and MFN2 might interact with OPA1, do the authors have any data in changes of MFN1 and OPA1 levels in this patient?

**Minor points:**

1. A cartoon to illustrate the main findings would be welcome. 
2. Sentence on page 4, paragraph 4, line 3, please rephrase (“Central nervous … of cases”)
3. Please state the glucose content of the medium during cell maintenance.
4. Page 5, third paragraph, line 9, goat anti-rabbit, should be goat anti-mouse.
5. Please state the company of the DAPI labelling.
6. Please state which program is used to do the statistical testing and if a p-value of <0.05 was considered significant.

Is the work clearly and accurately presented and does it cite the current literature?

Partly

If applicable, is the statistical analysis and its interpretation appropriate?

Partly

Are all the source data underlying the results available to ensure full reproducibility?

Yes

Is the study design appropriate and is the work technically sound?

Yes

Are the conclusions drawn adequately supported by the results?

Partly

Are sufficient details of methods and analysis provided to allow replication by others?

Yes

Reviewer Expertise:

I have worked on mitochondrial genetics for 3 decades, and now on mitochondrial dynamics and mitophagy.

We confirm that we have read this submission and believe that we have an appropriate level of expertise to confirm that it is of an acceptable scientific standard, however we have significant reservations, as outlined above.

## References

[1] : Distinct fission signatures predict mitochondrial degradation or biogenesis. *Nature* .2021;593(7859) : 10.1038/s41586-021-03510-6 435-439 10.1038/s41586-021-03510-6 33953403

[2] : Dysregulated mitophagy and mitochondrial organization in optic atrophy due to OPA1 mutations. *Neurology* .2017;88(2) : 10.1212/WNL.0000000000003491 131-142 10.1212/WNL.0000000000003491 27974645 PMC5224718

[3] : When MFN2 (mitofusin 2) met autophagy: A new age for old muscles. *Autophagy* .12(11) : 10.1080/15548627.2016.1215383 2250-2251 10.1080/15548627.2016.1215383 27575337 PMC5103344

# Response

**Published:** 2022-07-30

**Authors:** Timothy Shutt

**Affiliations:** University of Calgary, Canada

*>We would like to thank the reviewers for their interest in the manuscript and their suggestions, which we have taken to heart and have addressed to the best of our ability. We would also like to apologize for the extended period of time for the revisions. The first author left the lab shortly after the paper was initially submitted, and it took some time to get a new person up and running on the project after the reviews were all received. In addition to several clarifications in the text, the revised version of the manuscript contains new data showing alterations to the expression of mitochondrial fusion proteins, as well as new mtDNA copy number data normalized to B-tubulin. We hope that the revisions answer the questions and issues raised by the reviewers. Below are the specific point by point response to the reviews.*

 Reviewer 1

 Thank you for asking me to review this paper characterizing a mutation in MFN2 that results in an unusual phenotype. I think that this paper is ready for indexing provided they can confirm some additional details including the number of controls studied.

**Main points:**

 1. Neither the methods nor the results clearly spell out the N for each experiment. Figure 2B suggests that data was generated from 24 control and 27 patient fibroblasts.  How many controls were used? 

*>We have updated the figure legends to indicate the number of cells used for analysis in each experiment. Furthermore, all of the images analyzed are included in the extended data, should anyone wish to reanalyze the data.*

 The study would not be ready for publication if they only used one control. 

*>While we only used a single control line for these experiments, our previously published work has shown that this control line behaves similar to other control lines (PMID: 31231018). We have added the following text to make this clear to the reader:*

*“As a control fibroblast line, we used an age-matched cell line that we have shown previously to behave similar to other controls fibroblast lines with respect to mitochondrial function (PMID: 31231018).”*

 They should state how many repetitions of each of the experiments there were (their single statement, “…three independent biological replicates” on page 6 is not sufficiently detailed). These additional data would provide important support for the findings.

*>We have clarified the text in the methods as follows:*

*“For all of the image analysis in this manuscript, the data shown in the figures are from a single biological replicate with three technical replicates (e.g. images used for analysis were gathered from three coverslips processed in parallel). Experiments were repeated independently at least twice, for a total of three biological replicates, and all reported trends were consistent with the representative data reported in the figures.”*

 Even though the findings are relevant and interesting, that only one patient has been used should be stated more clearly along with the need to study more patients/relative with the novel D414V MFN2 variant.

*>We agree that more patients with the D414V variant would strengthen the study, unfortunately we are only aware of this single case with this clinical presentation. Other family members were not available for investigation and there was no family history of similar disease. We have clarified the text to highlight the fact that we have only one patient with the following addition to the discussion:*

*“Unfortunately, the D414V variant has not been described to date in any other patients. As such, we only have a single patient cell line in which to study this variant.”*

 2. The loss of mitochondria ER contacts (MERCs) is very beautiful and interesting.  This may well be linked to the slight reduction in mitochondrial DNA copy number and in respiratory chain function. The authors discuss the hypothesis that mtDNA replication is impaired in MFN2 because of the effect of excessive mitochondrial fragmentation on the distribution of the replication machinery (ref 19).  However, they barely discuss two other factors that are relevant to mtDNA replication: mitochondrial dynamics affect both nucleoid division and mitophagy (and hence mtDNA turnover).  Interactions with the ER are thought to license mtDNA replication as well as initiating nucleoid division (ref 30) so having a reduced MERC area, and potentially having a smaller midzone (Kleele *et al. *(2021 ^1^ )) could have an important effect. Impaired mitochondrial fusion may increase (Liao *et al*. (2017 ^2^ )) or decrease (Sebastián *et al*.(2016 ^3^ )) mitophagy in impaired MFN2 expression. Increased mitophagy could decrease mtDNA copy number and slow recovery from mtDNA depletion (ref 19).

*>As the initial discussion was already somewhat lengthy, we had not gone into details on these other possibilities. However, in light of the new data in the revised manuscript, which suggest that mtDNA copy number may be reduced in part by reduced levels of mitochondria, we have amended the discussion to further discuss other factors linking fusion to altered mtDNA.*

 3. The decrease in lipid droplets is interesting and I think novel in MFN2 mutants. Examining the distribution of lipid droplets in the form of distance to the nucleus is also a nice addition to the data. They should mention that (i) the exact molecular mechanisms how MFN2 maintains lipid homeostasis is unknown (ii) perilipin 1 much more abundant in adipose tissue (ref 34) than fibroblasts. From the discussion it is not clear how perilipins can explain the altered distribution found in the fibroblasts in the present paper. The lipid droplets in figure 5 could follow either (i) the mitochondrial network which seems also more perinuclear in the representative figure or (ii) the microtubular network which could be clarified by drug treatments such as nocodazole.

*>We appreciate the comments from the reviewer regarding the interesting aspects of lipid droplet alterations. We agree that there is certainly much more to learn in this regard, however, we believe that it is beyond the scope of the current investigation. As suggested, we included the following statements in our discussion:*

*“Though it should be noted that perilipin 1 is more abundant in adipose tissue that in fibroblasts (34).”*

*“… it is clear that the roles of MFN2 in maintaining lipid homeostasis are important, though the exact molecular mechanisms remain undefined”*

 4. Was the number of mitochondrial nucleoids or lipid droplets corrected for the cell size?

*>No, we did not correct for cell size, as we did not have a marker for the plasma membrane. Furthermore, the surface area covered by the cell does not necessarily correlate to the cell size if the cells do not attach and spread out to the same extent.*

 Did the authors also measure the size of the lipid droplets in these cells?

*>We did not measure the size of the lipid droplets, as the image processing required to visualize individual lipid droplets did not preserve the size information, though it is still reliable to quantify the number and distribution of the lipid droplets. At least by eye, the lipid droplet size does not appear to change between the cell lines.*

 5. The patient’s presentation is interesting: impaired vision, poor motor function and deafness. Of these, unusual features included early loss of vision, cerebellar ataxia, and brain MRI abnormalities.  However, some basic clinical details are missing.

- a) No segregation analysis is presented, presumably his parents may be no longer available. It would be useful to support recessive inheritance by identifying an asymptomatic individual in the pedigree who is heterozygous for the same mutation.

*>Indeed, the patient’s parents are not available for research, which is unfortunately common when investigating patients in adulthood. The suggestion to study heterozygous family members is of interest and we would have pursued this if other family members were available for research. Participation in research was offered to his sister who lives in the same city (who would have had a 50% chance of being a heterozygous carrier of this variant) but she declined participation. *

- b)  Analysis of skeletal muscle for mitochondrial DNA copy number and variable deletions.  Muscle is more relevant than these data that they present in fibroblasts, and should be available because the patient had a muscle biopsy.

*>Unfortunately, the muscle tissue was not available for mtDNA copy number analysis, as the patient’s muscle biopsy was done when he lived in a different country.*

 6. The authors present a nice visualization of mitochondrial fusion. As MFN1 is also important in mitochondrial fusion and MFN2 might interact with OPA1, do the authors have any data in changes of MFN1 and OPA1 levels in this patient?

*>At the reviewer’s suggestion, we investigated the levels of MFN1 and OPA1 in our patient fibroblasts, and discovered some interesting differences (Fig 1E). Notably, we see decreased protein levels for both MFN1 and MFN2, as well as several other mitochondrial proteins, suggesting that overall mitochondrial abundance is reduced in the patient cells. In addition, we see unexpected changes to the abundance of OPA1 protein isoforms, which to our knowledge have not been noted for other pathogenic MFN2 variants. These new data and a discussion of these novel results has been added to the revised manuscript.*

**Minor points:**

1. A cartoon to illustrate the main findings would be welcome. 
*>We decided against including a cartoon figure, as it is not simple to illustrate the various findings of this manuscript in a straightforward figure that is easy to interpret, and did not feel it would add significantly to the manuscript.*
2. Sentence on page 4, paragraph 4, line 3, please rephrase (“Central nervous … of cases”)
*>We have revised the text to make the meaning clearer, as follows: *

*“Central nervous system involvement is also rarely described (50), with periventricular and subcortical white matter lesions in 8 out of 21 patients from one series. A small minority of cases had transient neurological deficits such as dysarthria or paraesthesiae.”*

1. Please state the glucose content of the medium during cell maintenance.
*> The medium contains low glucose (5.56mM).*
2. Page 5, third paragraph, line 9, goat anti-rabbit, should be goat anti-mouse.
*> Corrected*
3. Please state the company of the DAPI labelling.
*>The DAPI used in this study is included in the Prolong Glass Antifade Mountant with NucBlue Stain from Fisher Scientific (Thermo Scientific P36983). In this case, the NucBlue in the commercial description is DAPI.*
4. Please state which program is used to do the statistical testing and if a p-value of <0.05 was considered significant.
*>All statistical analyses were performed using Prism, and yes, for all analyses the significant differences indicate p<0.05.*
